# Identification of diverse RNA viruses in *Obscuromonas* flagellates (Euglenozoa: Trypanosomatidae: Blastocrithidiinae)

**DOI:** 10.1093/ve/veae037

**Published:** 2024-05-04

**Authors:** Danyil Grybchuk, Arnau Galan, Donnamae Klocek, Diego H Macedo, Yuri I Wolf, Jan Votýpka, Anzhelika Butenko, Julius Lukeš, Uri Neri, Kristína Záhonová, Alexei Yu Kostygov, Eugene V Koonin, Vyacheslav Yurchenko

**Affiliations:** Life Science Research Centre, Faculty of Science, University of Ostrava, Ostrava 710 00, Czechia; Central European Institute of Technology, Masaryk University, Brno 625 00, Czechia; Life Science Research Centre, Faculty of Science, University of Ostrava, Ostrava 710 00, Czechia; Life Science Research Centre, Faculty of Science, University of Ostrava, Ostrava 710 00, Czechia; Life Science Research Centre, Faculty of Science, University of Ostrava, Ostrava 710 00, Czechia; National Center for Biotechnology Information, NLM, National Institutes of Health, Bethesda 20894, USA; Institute of Parasitology, Biology Centre, Czech Academy of Sciences, České Budějovice 370 05, Czechia; Department of Parasitology, Faculty of Science, Charles University, Prague 128 00, Czechia; Life Science Research Centre, Faculty of Science, University of Ostrava, Ostrava 710 00, Czechia; Institute of Parasitology, Biology Centre, Czech Academy of Sciences, České Budějovice 370 05, Czechia; Faculty of Science, University of South Bohemia, České Budějovice 370 05, Czechia; Institute of Parasitology, Biology Centre, Czech Academy of Sciences, České Budějovice 370 05, Czechia; Faculty of Science, University of South Bohemia, České Budějovice 370 05, Czechia; The Shmunis School of Biomedicine and Cancer Research, Tel Aviv University, Tel Aviv 39040, Israel; Life Science Research Centre, Faculty of Science, University of Ostrava, Ostrava 710 00, Czechia; Institute of Parasitology, Biology Centre, Czech Academy of Sciences, České Budějovice 370 05, Czechia; Department of Parasitology, Faculty of Science, Charles University, BIOCEV, Vestec 252 50, Czechia; Division of Infectious Diseases, Department of Medicine, University of Alberta, Edmonton, Alberta T6G 2G3, Canada; Life Science Research Centre, Faculty of Science, University of Ostrava, Ostrava 710 00, Czechia; Zoological Institute of the Ruian Academy of Sciences, St. Petersburg 199034, Russia; National Center for Biotechnology Information, NLM, National Institutes of Health, Bethesda 20894, USA; Life Science Research Centre, Faculty of Science, University of Ostrava, Ostrava 710 00, Czechia

**Keywords:** *Obscuromonas*, *Blastocrithidia*, dsRNA viruses, *Narnaviridae*, *Mitoviridae*, Qin-like virus

## Abstract

Trypanosomatids (Euglenozoa) are a diverse group of unicellular flagellates predominately infecting insects (monoxenous species) or circulating between insects and vertebrates or plants (dixenous species). Monoxenous trypanosomatids harbor a wide range of RNA viruses belonging to the families *Narnaviridae, Totiviridae*, *Qinviridae*, *Leishbuviridae,* and a putative group of tombus-like viruses. Here, we focus on the subfamily Blastocrithidiinae, a previously unexplored divergent group of monoxenous trypanosomatids comprising two related genera: *Obscuromonas* and *Blastocrithidia*. Members of the genus *Blastocrithidia* employ a unique genetic code, in which all three stop codons are repurposed to encode amino acids, with TAA also used to terminate translation. *Obscuromonas* isolates studied here bear viruses of three families: *Narnaviridae, Qinviridae*, and *Mitoviridae*. The latter viral group is documented in trypanosomatid flagellates for the first time. While other known mitoviruses replicate in the mitochondria, those of trypanosomatids appear to reside in the cytoplasm. Although no RNA viruses were detected in *Blastocrithidia* spp., we identified an endogenous viral element in the genome of *B. triatomae* indicating its past encounter(s) with tombus-like viruses.

## Introduction

Trypanosomatids (Euglenozoa: Kinetoplastea: Trypanosomatidae) are unicellular flagellates that parasitize various invertebrates, vertebrates, and plants (Kostygov et al. 2021b). The majority of trypanosomatid genera are monoxenous, i.e. restricted to only one host (predominantly an insect), but some are dixenous, i.e. they alternate between two hosts and frequently cause severe diseases in vertebrates and plants (Stuart et al. 2008; Frolov, Kostygov, and Yurchenko 2021). Trypanosomatids constitute a ‘playground’ for evolution as they harbor numerous innovations (Kostygov et al. 2024). These include massive uridine insertion/deletion editing of mitochondrial mRNAs (Aphasizheva et al. 2020; Lukeš, Kaur, and Speijer 2021), peroxisome-compartmentalized carbohydrate metabolism (Gabaldón, Ginger, and Michels 2016), lack of transcriptional regulation for protein-coding genes (Clayton 2019), and *trans*-splicing of nuclear mRNAs (Michaeli 2011), to mention only a few prominent features.

One of the most unusual features discovered in trypanosomatids is the deviation from the standard nuclear genetic code observed in *Blastocrithidia* spp., in which all three stop codons have been reassigned to encode amino acids (aa) (Záhonová et al. 2016). In members of this genus, two tRNA^Glu^ are cognate for UAA and UAG and a uniquely shortened (4 bp versus the ubiquitous 5 bp anticodon stem) tRNA^Trp^ allows its reassignment to UGA. The UAA codon also serves as the sole translation terminator (Kachale et al. 2023). Notably, all members of the phylogenetically related genus *Obscuromonas* adhere to the standard nuclear genetic code. The widespread nonsense-mediated mRNA decay pathway responsible for the degradation of mRNAs containing premature stop codons (Baker and Parker 2004) is absent from both genera and appears to be a prerequisite for the genetic code switch in *Blastocrithidia* (Nenarokova et al. 2019). The availability of two phylogenetically close taxa with different genetic codes provides a unique opportunity for comparative analysis aimed at understanding the factors and consequences of genetic code alteration.

Ever since the first observation of virus-like particles in *Leishmania hertigi* (Molyneux 1974) and molecular characterization of *Leishmania RNA virus 1* in *L. guyanensis* (Stuart et al. 1992), trypanosomatids were shown to host diverse RNA viruses (Grybchuk et al. 2018b). So far, six RNA virus families infecting Trypanosomatidae have been identified, with at least three of them (*Leishbuviridae, Narnaviridae*, and *Totiviridae*) detected in more than one host lineage (Grybchuk et al. 2018a, 2018c, 2020; Klocek et al. 2023; Macedo et al. 2023). Viruses of these and related families are frequently found in meta-transcriptomes of arthropods as well as in various fungi and oomycetes (Lee Marzano et al. 2016; Schoonvaere et al. 2018; Batson et al. 2021). They comprise a non-taxonomical ecological group of viruses that persist (often asymptomatically) in protistan or fungal cells (Ghabrial et al. 2015; Hough et al. 2023). Some members of *Totiviridae* and *Mitoviridae* attenuate virulence in phytopathogenic fungi (Ghabrial and Suzuki 2009; Wu et al. 2010). The diversity and various features of trypanosomatid viruses were recently reviewed (Cantanhêde et al. 2021; Kostygov et al. 2021a).

Both *Narnaviridae* and *Mitoviridae* are capsid-less positive-sense single-stranded RNA (+ssRNA) viruses of the phylum *Lenarviricota* initially described in fungi and, subsequently, in other hosts (Matsumoto, Fishel, and Wickner 1990; Polashock and Hillman 1994; Ghabrial et al. 2015). The defining feature of mitoviruses and narnaviruses is the presence of a single large open reading frame (ORF) encoding the RNA-dependent RNA polymerase (RdRP), which is phylogenetically related to that of RNA bacteriophages of the family *Fiersviridae* (formerly Leviviri*dae*). The phylum *Lenarviricota* is the basal lineage with respect to all other RNA viruses of the kingdom *Orthornavirae* (Wolf et al. 2018, 2020). Based on the codon usage and GC content, mitoviruses are thought to replicate in the host mitochondria. In contrast, narnaviruses, which apparently evolved from mitoviruses, replicate in the cytoplasm (Hillman and Cai 2013; Nibert 2017). It is generally assumed that mitoviruses originated from a fiersvirus that replicated in the alphaproteobacterial proto-mitochondrial endosymbiont and persisted within mitochondria during eukaryogenesis (Koonin, Dolja, and Krupovic 2015). This lifestyle implies adaptation to the mitochondrial translation system, i.e. the use of mitochondrial genetic code.

In this work, we analyzed the viromes of several members of the subfamily Blastocrithidiinae and documented various RNA viruses in *Obscuromonas* spp. In contrast, no RNA viruses were detected in three studied isolates of *Blastocrithidia* spp. although the nuclear genome of *Blastocrithidia triatomae* was shown to contain an endogenous viral element (EVE) originating from a tombus-like virus.

## Results

### Screening of Blastocrithidiinae for RNA viruses

**Figure 1.**
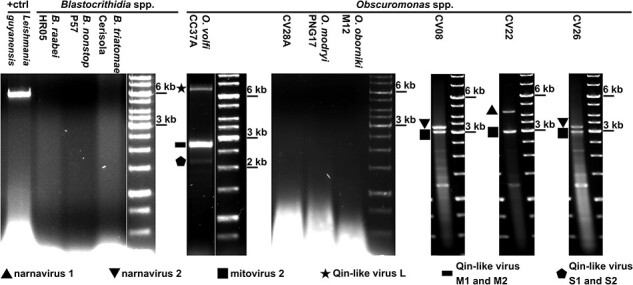
dsRNA screening in *Obscuromonas* spp. and *Blastocrithidia* spp. *Leishmania guyanensis* infected with *Leishmania RNA virus 1* (LRV1) was used as a positive control. Molecular weight marker sizes are indicated on the right. Species names and IDs of the analyzed isolates (Table 1) are shown on top.

Eighteen isolates of seven *Obscuromonas* spp. and three isolates representing different species of *Blastocrithidia* were analyzed in agarose gels for the presence of double-stranded RNA (dsRNA) that is indicative of RNA viruses (Fig. 1, Table 1). Only five isolates of *Obscuromonas* spp. were found to be virus-positive. No RNA viruses were identified in the examined *Blastocrithidia* spp. isolates either by agarose gel electrophoresis (Fig. 1) or by high-throughput sequencing (HTS), which effectively excludes the possibility that some viruses were not detected because of their low abundance. All viral contigs, except the partial narnaviral sequence in the isolate CV01 that was considered sequence contamination, had higher coverage (192–2,927 Reads Per Kilobase per Million mapped reads (RPKM), mean 1,019) compared to the protein-coding contigs (12–736 RPKM, mean 75). The most numerous and abundant classes of protein-coding RNAs were of viral or trypanosomatid origin suggesting no contamination with other protists (Supplementary Data S1).

**Table 1.** List of screened isolates of Blastocrithidia and Obscuromonas spp.

Species	Isolate	Virus	Segment	Size, nt	ORF, aa	RPKM	Accession
*Obscuromonas* sp. 1	CV03	Narnavirus 1	L	3,764	1,217	845.1	OR723805
*Obscuromonas* sp. 1	CV08	Narnavirus 2	L	2,999	835	736.5	OR723802
		mito1	L	2,643	703	312.7	OR723807
		mito2	L	2,649	703	438.0	OR723810
*Obscuromonas* sp. 1	CV22	Narnavirus 1	L	3,767	1,217	785.5	OR723806
		mito2	L	2,646	703	2927.1	OR723809
*Obscuromonas* sp. 1	CV26	Narnavirus 2	L	2,999	835	1911.5	OR723801
		mito1	L	2,875	703	2918.5	OR723808
*Obscuromonas volfi*	CC37A	Qin-like	L	6,162	1,937	333.9	OR723812
			M1	2,537	641	302.2	PP502392
			M2	2,521	623	192.0	PP502393
			S1	2,015	423	524.1	OR723813
			S2	2,078	633	111.4	PP534172
*Obscuromonas* sp. 1	CV01	–					
*Obscuromonas* sp. 1	CV04	–					
*Obscuromonas* sp. 1	CV05	–					
*Obscuromonas* sp. 2	CV15	–					
*Obscuromonas* sp. 1	CV17	–					
*Obscuromonas* sp. 3	CV27	–					
*Obscuromonas* sp. 3	CV28A	–					
*Obscuromonas* sp. 4	CC49A	–					
*Obscuromonas oborniki*	M9	–					
*Obscuromonas oborniki*	M12	–					
*Obscuromonas modryi*	PNG17	–					
*Obscuromonas modryi*	FI15	–					
*Obscuromonas modryi*	PNG106	–					
*Blastocrithidia nonstop*	P57	–					
*Blastocrithidia raabei*	HR05	–					
*Blastocrithidia triatomae*	Cerisola	–					

### RNA viruses of Obscuromonas

HTS analyses revealed three types of RNA viruses in *Obscuromonas* spp.: (1) mitoviruses (2.7 kb), (2) two different narnaviruses (3.0 and 3.8 kb), and (3) a Qin-like virus (five segments of 6.2, 2.5, 2.5, 2.0, and 2.0 kb) (Fig. 1, Table 1). Several viruses were found to infect the same isolate. In most cases, isolates were mono-specific except for *Obscuromonas volfi* CC37A, which turned out to contain another species of the same genus (*Obscuromonas* sp. 1, as judged by a considerable proportion of its 18S rRNA gene reads in the HTS data). It was cloned as described elsewhere (Hamilton et al. 2015) and reanalyzed.

**Figure 2.**
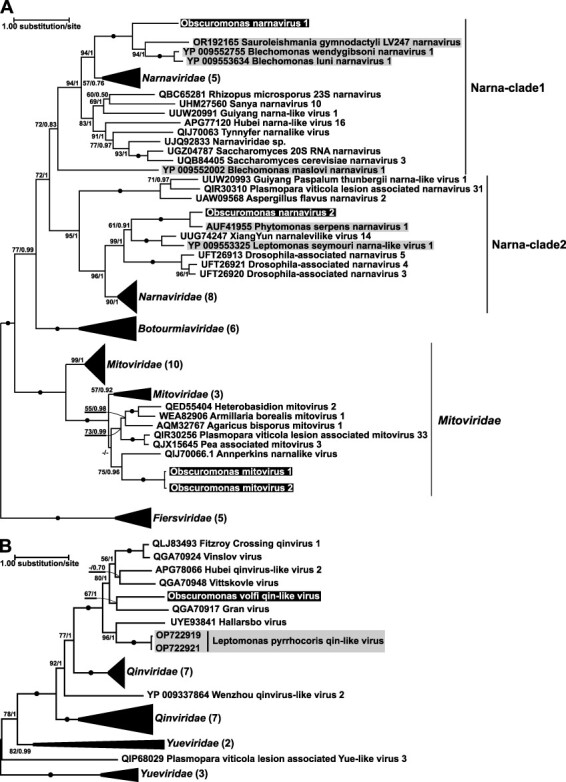
Maximum likelihood phylogenetic trees based on viral RdRP amino acid sequences from *Obscuromonas* spp. (A) The tree for *Narnaviridae* and *Mitoviridae* rooted with *Fiersviridae*; two major clades of narnaviruses are indicated with vertical lines. (B) The tree for *Qinviridae* rooted with Yue-like viruses. Some clades in (A) and (B) were collapsed for simplicity. Numbers at branches represent standard bootstrap replicates and Bayesian posterior probabilities. Absolute supports (100/1) are shown with solid circles, those below 50 or 0.5 are replaced with dashes. The sequences of trypanosomatid viruses reported in this and in previous studies are highlighted in black and gray, respectively. For full trees in the Newick format, see Data S2 and S3.

Mitoviruses were found in three out of five positive isolates (Table 1). Their sequences were highly similar at the nucleotide level (91.2–99.3 per cent identity). Phylogenetic analysis based on the RdRP aa sequence confidently placed these viruses within the clade of *Mitoviridae*, distinct from narnaviruses of *Obscuromonas* and other trypanosomatids (Fig. 2A). These mitoviruses formed a robustly supported clade, which we consider to be a single species, with two subclades, each composed of identical aa sequences (Fig. 2A). Interestingly, the isolate CV08 harbored viruses from both subclades (Table 1).

Two species of narnaviruses were detected in four of the five positive isolates of *Obscuromonas* spp. Nucleotide sequences of each narnavirus species were highly conserved. In the RdRP-based phylogenetic tree, these two species were nested within the two major narnaviral clades and proved to be the closest relatives of viruses previously described from four other trypanosomatid taxa: *Blechomonas, Leishmania* (*Sauroleishmania*), *Leptomonas*, and *Phytomonas* (Fig. 2A) (Kraeva et al. 2015; Akopyants et al. 2016; Grybchuk et al. 2018c; Klocek et al. 2023). Although co-infections by two narnaviruses were not observed, mitoviruses were invariably accompanied by a narnavirus (Table 1).

The only occurrence of a Qin-like virus was documented in the isolate CC37A and it was the sole virus infecting this isolate (Fig. 1, Table 1). This family of negative sense RNA viruses is rare in trypanosomatids and was previously detected only in *Leptomonas pyrrhocoris* (Macedo et al. 2023). BLAST searches with assembled HTS contigs of CC37A dsRNA (from this work) against UniRef50 identified three genomic segments of a novel Qin-like virus. The large segment (6.2 kb) contained an ORF encoding RdRP of 1,937 aa, the middle segment (denoted M1, 2.5 kb) harbored an ORF coding for a putative envelope glycoprotein (641 aa) homologous to that of *Leptomonas pyrrhocoris leishbunyavirus 4* (LBV4, GenBank accession number WMB96339, the only BLAST-hit, 24 per cent aa sequence identity), whereas the small segment (denoted S1, 2 kb) comprised an ORF encoding a 423 aa-long protein that shared 20 per cent aa sequence identity with a hypothetical protein of the *Wenzhou qinvirus-like virus 1* (GenBank accession number YP_009337867). Two additional identified genomic segments (middle, denoted M2, and small, denoted S2; 2.5- and 2.0-kb-long, respectively) were found to have similar coverage to that of other three viral segments and contained ORFs spanning 74 and 91 per cent of the segment length, respectively (Table 1). These ORFs code for hypothetical proteins (623 and 633 aa in length) with no detectable homologs in the current sequence databases. Superposition of the two 2.5-kb-long genomic segments explains the higher relative brightness of the corresponding band in the agarose gel (Fig. 1), even though all viral segments have similar coverage in the range between 200 and 500 RPKM. The putative glycoprotein encoded in the M1 segment contains conserved hydrophobic patches and cysteine residues shared with LBV4 and other *Phenuiviridae*. Phylogenetic reconstruction revealed monophyly of of glycoproteins from LBV4 and Qin-like viruses with absolute statistical support. despite a long evolutionary distance between the latter (Supplementary figure S1). The RdRP-based phylogeny showed that this virus belongs to a clade that also contains meta-transcriptomic sequences from mosquitoes (likely, viruses of trypanosomatids from these insects) and not directly related to the Qin-like virus of *L. pyrrhocoris* (Fig. 2B).

### An EVE in *Blastocrithidia*

**Figure 3.**
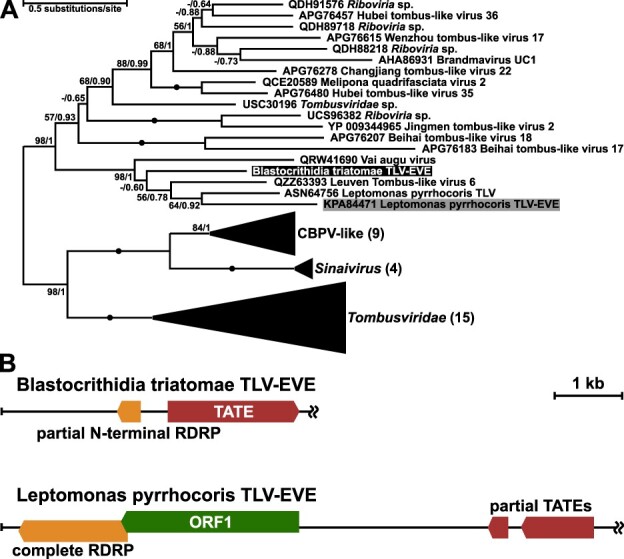
EVE of *Blastocrithidia triatomae*. (A) Maximum likelihood phylogenetic tree based on RdRP amino acid sequences of viruses and EVEs. Numbers at branches represent standard bootstrap replicates and Bayesian posterior probabilities. Absolute supports (100/1) are shown with solid circles, those below 50 or 0.5 are replaced with dashes. *Blastocrithidia triatomae* EVE is highlighted in black, *Leptomonas pyrrhocoris* EVE—in gray. For full tree in the Newick format, see Data S4. (B) Position of EVEs in genomes of *B. triatomae* and *L. pyrrhocoris*. Double wiggly lines indicate continuation of the scaffold (chromosome).

Although RNA viruses were not found in *Blastocrithidia* spp., we detected an EVE integrated into the genome of *B. triatomae* (Fig. 3) and named it *Blastocrithidia triatomae* tombus-like virus EVE (*Btri*TLV-EVE). No such element was detected in the genomes of other two *Blastocrithidia* spp. and all four *Obscuromonas* spp. sequenced. The *Btri*TLV-EVE contains a 119 codon-long partial ORF sharing 42 per cent aa identity with the N-terminal sequence of the RdRP of *L. pyrrhocoris* tombus-like virus (*Leppyr*TLV) (GenBank accession number ASN64759) (Grybchuk et al. 2018a) including the conserved F motif (Bruenn 2003). Phylogenetic analysis of this element showed its association with the clade containing *Leppyr*TLV and *Leppyr*TLV-EVE (Fig. 3A), although with no direct relationship with them. This suggests that TLVs in *L. pyrrhocoris* and *B. triatomae* were acquired independently. Notably, the *Btri*TLV-EVE used the same non-canonical genetic code as its host, with UAA, UAG, and UGA all encoding amino acids. Similarly to the *Leppyr*TLV-EVE in the genome of *L. pyrrhocoris* (Grybchuk et al. 2018a), the *Btri*TLV-EVE is located in the sub-telomeric region in the vicinity of a TATE DNA transposon (Fig. 3B). Additional BLAST searches in *B. triatomae* genome identified over 150 TATE sequences with nucleotide identity ranging from 79 to 98 per cent, but only one of them was associated with this EVE (Supplementary Table S1), which is compatible with the hypothesis of a spontaneous capture, reverse transcription and integration of a replicating viral RNA by the transposon.

## Discussion

In this work, we investigated RNA viruses of Blastocrithidiinae and found the viral content of *Obscuromonas* spp. to be unusual for trypanosomatids. None of the eighteen analyzed isolates harbored *Leishbuviridae*, the virus family most commonly encountered in monoxenous trypanosomatids (Grybchuk et al. 2018a, 2018c). Instead, the investigated flagellates were mostly infected by rarer narnaviruses and mitoviruses, the latter being documented in protists for the first time.

The UGA codon, which terminates translation in the cytoplasm, was repurposed to encode Trp in the mitochondria of most eukaryotes (Fox 1979; Uddin et al. 2020), including trypanosomatids (de la Cruz, Neckelmann, and Simpson 1984; Simpson et al. 2000; Záhonová et al. 2021), and many mitoviruses indeed employ this mitochondrial genetic code. However, mitoviruses of *Obscuromonas* do not encode Trp *via* UGA. Prior to this work, there were only two documented examples of such a mismatch between the codes used by a mitovirus and its host mitochondria, both from fungi of the phylum Ascomycota: a virus of *Erysiphe necator* (a single non-UGA mitovirus out of the twenty-seven viruses documented in this species) and a virus of *Neofusicoccum parvum* (a single non-UGA mitovirus of the three identified in this species) (Nerva et al. 2019). In addition, the GC content of the *Obscuromonas* mitoviruses (49 per cent) is considerably higher than that of mitochondrial genomes of their hosts (about 18 per cent on average in *O. modryi* (Albanaz et al. 2023)). Taken together, these two features suggest that the mitoviruses of *Obscuromonas* replicate in the cytoplasm rather than in the mitochondrion. This appears to be one of the rare cases of a mitovirus exiting into the cytoplasm, recapitulating the likely founding event, which led to the origin of narnaviruses. Based on the phylogenetic position of the mitoviruses from *Obscuromonas*, we hypothesize that they originated by the horizontal transfer of a cytoplasmic mitovirus from a fungus to trypanosomatids.

Our phylogenetic inferences demonstrate that trypanosomatids host viruses of both major lineages of *Narnaviridae*. Moreover, some narnaviruses from different trypanosomatids are closely related to each other although their relationships are not congruent with those of the flagellate hosts, that is, no obvious coevolution was detected. However, once adapted to trypanosomatids, these viruses apparently tend to stay confined to these hosts. Switching between trypanosomatid genera is then facilitated by their ecologically determined contacts as has been previously proposed for *Leishmania* (*Sauroleishmania*) and *Blechomonas* (Grybchuk et al. 2018c; Klocek et al. 2023) and further exemplified here by the newly discovered pair *Phytomonas—Obscuromonas* both parasitizing phytophagous bugs (Podlipaev 1990; Lukeš et al. 2021). While narnaviruses from two different clades can infect the same trypanosomatid species, they were never detected in a single isolate together, even though narnavirus and mitovirus co-infections were documented here. It appears likely that narnaviruses compete with each other for host resources, which leads to their mutual exclusion.

*Qinviridae* is an obscure family of -ssRNA viruses. So far, only trypanosomatids *Obscuromonas volfi* (this study) and *Leptomonas pyrrhocoris* (Macedo et al. 2023) were confirmed as hosts of these viruses. From the available metatranscriptomic data, it was inferred that qinviruses have two genomic segments even though their RdRp is closely related to those of the monosegmented—ssRNA viruses of the order *Mononegavirales* (Wolf et al. 2023). In this work, we obtained clonal lines of the Qin-like virus-bearing isolate *O. volfi* CC37A, which allowed us to directly visualize virus-enriched dsRNAs in agarose gel and identify three previously uncharacterized viral genomic segments. It is unclear whether this genome arrangement with five fragments is a common, but overlooked, feature of all *Qinviridae* or a peculiarity of this particular virus. The fact that *Qinviridae* and *Leishbuviridae* that belong to different orders of −ssRNA viruses, might co-occur in the same hosts (Trypanosomatidae) raises an intriguing possibility of genomic segment reassortment between distantly related viruses, similar to the apparent cross-phylum reassortment event in *Ourmiavirus* (Rastgou et al. 2009). And we apparently have an evidence for this: the glycoprotein of the qinvirus from *Obscuromonas volfi* has a leshbunyaviral origin.

The small sample size constrained by the availability of *Blastocrithidia* isolates in culture precludes us from making a conclusion on the occurrence of viruses in these flagellates. Nevertheless, the lack of detected viruses is consistent with the hypothesis that the altered genetic code of *Blastocrithidia* spp. serves as an antiviral barrier that protects its bearers from viral infections and gene transfer (Holmes 2009; Taylor and Bruenn 2009; Lajoie et al. 2013; Taylor et al. 2013; Ostrov et al. 2016). However, this barrier does not seem to be impenetrable as indicated by the finding of a recoded EVE in the *B. triatomae* genome, suggesting that in the past this species or its recent ancestor harbored an RNA virus related to *Tombusviridae*. This TLV must have survived the suboptimal conditions in the new host and, apparently, adopted the altered code before integrating into the host genome. The code switch would be a near dead-end in virus evolution, whereby the virus would lose the ability to infect hosts with the canonical genetic code and would remain ‘locked’ in members of the genus *Blastocrithidia*. Nonetheless, many clades of RNA viruses, some at and beyond family level, display evidence of using alternative genetic codes (Wolf et al. 2020; Neri et al. 2022; Nyerges et al. 2023). Thus, despite the obvious limitations imposed on viral spread by the host lock-in due to the use of a non-canonical genetic code, some RNA viruses appear to have adapted to reproduction in such hosts.

The only other trypanosomatid, in which an EVE was discovered (along with its cognate TLV), is *L. pyrrhocoris*, which belongs to a different trypanosomatid subfamily, Leishmaniinae (Grybchuk et al. 2018a). Based on the phylogenetic distances between the trypanosomatids and their TLV EVEs, they appear to have acquired the TLVs independently. Acquisition of viruses in both cases could have been facilitated by the feeding habits of the insect hosts of the respective trypanosomatids. Because of the extensive degradation of *Btri*TLV-EVE, it seems likely that the corresponding virus was acquired by the ancestor of *B. triatomae* earlier on the evolutionary scale than the TLV-EVE of *L. pyrrhocoris*.

In conclusion, RNA viruses of Blastocrithidiinae differ from those previously described in other trypanosomatids and represent a promising data set for further comparative studies of viruses infecting related hosts with canonical and non-canonical genetic codes. Analysis of the genomes and viromes of additional species and isolates of *Blastocrithidia* and *Obscuromonas* should help in understanding the potential antiviral role of the altered genetic codes and the restrictions that code switches can impose on RNA virus evolution.

## Materials and methods

### Cultivation, dsRNA extraction, and sequencing

Axenic cultures of *Obscuromonas* spp. and *Blastocrithidia* spp. were grown in the Schneider’s *Drosophila* Medium (Thermo Fisher Scientific, Waltham, USA) supplemented with 10 per cent fetal bovine serum (Sigma-Aldrich/ Merck, St. Louis, USA), 10 µg/ml of hemin (Jena Bioscience, Jena, Germany), 500 units/ml of penicillin, and 0.5 mg/ml of streptomycin (both Thermo Fisher Scientific). Species identity was confirmed as previously (Yurchenko et al. 2016).

Total RNA was isolated from 10^9^ cells with TRI reagent (Molecular Research Center, Cincinnati, USA). For screening, 50 µg of total RNA was treated with DNase I and S1 nuclease (Thermo Fisher Scientific and Sigma-Aldrich/ Merck, respectively) for 1 h at 37°C to remove DNA and single-stranded RNA as described previously (Grybchuk et al. 2018a). The resulting samples were resolved on a 0.8 per cent agarose gel and post-stained with Midori green (Nippon Genetics Europe, Düren, Germany) as in Klocek et al. (2023). For sequencing, 400 µg of total RNA from the selected samples was digested with DNase I/ S1 nuclease and purified using Zymoclean Gel RNA recovery kit (Zymo Research, Irvine, USA). The RiboMinus libraries were sequenced using Illumina NovaSeq 6000 (Illumina, San Diego, USA) at Macrogen Europe (Amsterdam, Netherlands) or the Institute of Applied Biotechnologies (Olomouc, Czechia).

### HTS data processing

Paired-end reads were trimmed and quality-filtered with BBDuk of BBTools v. 39.00 (Bushnell, Rood, and Singer 2017), decontaminated against genomes of cellular organisms with BBmap, and assembled *de novo* using SPAdes v. 3.15.5 (Prjibelski et al. 2020). The obtained contigs were analyzed using DIAMOND v. 2.0.2 (Buchfink, Reuter, and Drost 2021) with the BLASTx algorithm and the UniRef50 database (Mirdita et al. 2017), and BLAST+ v. 2.13.0 (Camacho et al. 2009) with PSI-BLAST algorithm and six-frame translated contigs used as a query against database of viral RdRP profiles (Neri et al. 2022). False positives were identified by BLASTp searches of recovered contig translations against the NCBI non-redundant (nr) database (download date: 20 February 2023). The remaining contigs were used for BLASTn and BLASTx searches as above against NCBI nucleotide (download date: 8 May 2022) and UniClust50 (download date: 24 December 2023) databases, respectively. To compute contig coverage, reads were mapped to contigs using Bowtie 2 v. 2.4.4 (Langmead and Salzberg 2012) and sorted with SAMtools v. 1.13 (Danecek et al. 2021). Numbers of reads covering each contig were analyzed in BEDTools v. 2.30.0 (Quinlan 2014). From the latter value, the RPKMs were calculated. The data on contigs’ length, coverage, and identity were integrated using custom *bash* script. Contigs of at least 750 nt in length with homologs in the UniClust database were split into groups based on their taxonomy and the number of contigs and their average RPKM were calculated for each group (Data S1).

### Blastocrithidia triatomae genome assembly

Total genomic DNA of *B. triatomae* was isolated from ∼10^8^ cells using the DNeasy Blood and Tissue Kit (Qiagen, Hilden, Germany) according to the manufacturer’s protocol. The genome was sequenced using Illumina NovaSeq 6000 (performed by Macrogen Inc., Seoul, The Republic of Korea). Paired-end reads were quality and adapter trimmed using BBDuk of BBTools v. 38.98. To obtain the best assembly, we applied three strategies: (1) using reads with a minimum length of 75 nt, error-corrected by Karect (Allam, Kalnis, and Solovyev 2015) and *de novo* assembled by SPAdes v. 3.13.0 without the error correction step; (2) using reads with a minimum length of 75 nt, *de novo* assembled by SPAdes with an error correction step (using careful option); and (3) using all reads *de novo* assembled by SPAdes with error correction step (using careful option). Assembly (3) proved to be the best (Supplementary Table S2) according to QUAST v. 5.2.0 metrics (Mikheenko et al. 2018). The assembly was further improved by two rounds of scaffolding using Platanus v. 1.2.4 (Kajitani et al. 2014) and gap-filling using GapCloser v. 1.12 from SOAPdenovo2 (Luo et al. 2012). Scaffolds shorter than 500 bp were removed, and the assembly was checked for a potential contamination by BlobTools v. 1.1.1 (Laetsch and Blaxter 2017). Scaffolds were screened by BLASTn against the National Center for Biotechnology Information (NCBI) nucleotide database, and those having sequence identity over 95 per cent and query coverage over 85 per cent to non-euglenozoan sequences were removed. Scaffolds with non-euglenozoan hits below the removal threshold were further screened against the nr database (download date: 14 June 2022) by DIAMOND v. 2.0.15 (Buchfink, Reuter, and Drost 2021) and kept if euglenozoan hits were retrieved. Final assembly statistics from QUAST are shown in Supplementary Table S2.

### Phylogenetic inference

Sequences of the mitoviral and narnaviral RdRPs from *Obscuromonas* spp. were used as queries for three iterations of PSI-BLAST search against the nr database. The retrieved homologous sequences were aligned using MAFFT v. 7.490 with the FFT2 algorithm (Katoh and Standley 2013); incomplete and redundant sequences (over 50 per cent pairwise identity) were removed using ‘reduce redundancy’ function in Jalview2 alignment viewer (Waterhouse et al. 2009). Datasets were then merged and duplicate sequences were removed. The final dataset was aligned with the MAFFT G-INS-i iterative algorithm with the maximum number of iterations set to 1,000. The resulting alignment was trimmed using trimAl v. 1.4 (Capella-Gutiérrez, Silla-Martinez, and Gabaldon 2009). Altogether, fourteen different gap thresholds (from 0.2 to 0.95) were tested and the parameter 0.5 resulted in the highest average ultrafast bootstrap support. The maximum likelihood (ML) and Bayesian trees were inferred using IQ-TREE v. 2.1.2 (Minh et al. 2020) and MrBayes v. 3.2.7a (Ronquist et al. 2012), respectively. The tree was rooted with *Fiersviridae* as in recent publications (Wolf et al. 2018, 2020). For ML, the best-fit model LG + F + I + G4 was selected by ModelFinder (Kalyaanamoorthy et al. 2017) and 1,000 standard bootstrap replicates were used for the estimation of branch support. For Bayesian inference, LG + F + I + G4 was set and posterior probability density sampling was run for 1,000,000 generations with default settings. The coding nucleotide sequences corresponding to aa sequences of mitoviruses were downloaded from the NCBI database and applied to analyze UGA codon usage.

RdRP sequences of Qin-like viruses were retrieved by PSI-BLAST search against the nr database as described previously (Macedo et al. 2023). Twenty-nine best matches including two recently described Qin-like viruses from *Leptomonas pyrrhocoris* were selected for analysis. The Yue-like viruses were included as the closest outgroup (Käfer et al. 2019). Sequences were aligned with the E-INS-i algorithm in MAFFT v. 7.520 (1,000 iterations) and trimmed in trimAl using ‘automated1’ heuristic algorithm. The ML and Bayesian trees were inferred as above.

Selection of sequences for phylogenetic reconstruction of tombus-like EVEs was based on the previously published trees (Macedo et al. 2023). Chronic bee paralysis virus, members of the genus *Sinaivirus,* and *Tombusviridae* spp. were added as outgroups for rooting. Sequences were aligned using MAFFT (G-INS-i) and trimmed by trimAl using the automated algorithm. The ML and Bayesian trees were inferred as above.

## Supplementary Material

veae037_Supp

## Data Availability

New sequences reported here (Table 1) are available from the GenBank under accession numbers OR723804-OR723813. The raw DNA reads of *B. triatomae* are available at NCBI under the BioProject PRJNA1011240. The raw RNA reads from this work are available at NCBI under the BioProject PRJNA1094563. All other data generated or analyzed during this study are included in this published article and its supplementary information files.
