# Supplementary material for: Identification of diverse RNA viruses in Obscuromonas flagellates (Euglenozoa: Trypanosomatidae: Blastocrithidiinae)
Source: Virus Evol. 2024 May 4;10(1):veae037. doi: 10.1093/ve/veae037 (PMC11108086; doi:10.1093/ve/veae037)
Supplement: veae037_Supp [file veae037_supp.zip › suppl_data/Data S1.docx]

Statistics of the top-20 most covered contigs based on the taxon reported in BLASTn and BLASTx searches.

Key

Taxon | number of contigs | average RPKM

TrypNT_cov = contigs with trypanosomatid BLASTN hits

rest = contigs with UniProt BLASTX hits

##########

CV08 (Narna, Mito)

##########

viral metagenome | 1 | 16154.9

TrypNT_cov | 2177 | 1043.27

Annperkins narna-like virus | 4 | 353.052

Crithidia sp. ATCC 30255 | 4 | 164.698

Trypanosoma | 1075 | 123.168

Leptomonas seymouri | 14 | 89.8083

cellular organisms | 18 | 84.4444

Trypanosoma grayi | 72 | 44.2284

Trichuris suis | 1 | 42.5454

Trypanosoma brucei | 10 | 41.9254

Mesangiospermae | 1 | 40.7813

Trypanosomatidae | 598 | 40.1438

Leishmania braziliensis MHOM/BR/75/M2904 | 94 | 39.7781

Pavlovaceae | 1 | 39.3192

Leptomonas pyrrhocoris | 390 | 38.9397

Leishmania braziliensis | 103 | 38.2708

Eukaryota | 56 | 36.3939

Leptomonas | 501 | 34.9175

Trypanosoma cruzi | 72 | 34.3883

Herpetomonas muscarum | 5 | 33.1018

##########

CV22 (Narna, Mito)

##########

Annperkins narna-like virus | 1 | 2927.14

Phytomonas serpens narnavirus | 1 | 785.539

TrypNT_cov | 2572 | 758.27

Leishmania donovani | 418 | 232.342

Leishmania | 1533 | 86.2375

Crithidia acanthocephali | 11 | 57.9435

Trypanosoma cruzi | 111 | 54.6192

Crithidia sp. ATCC 30255 | 17 | 48.2315

Trypanosoma cruzi marinkellei | 13 | 47.078

Trypanosoma grayi | 123 | 43.4744

Leishmania infantum | 64 | 43.3138

Eukaryota | 69 | 38.8731

Leishmania braziliensis | 148 | 38.1888

Leishmania braziliensis MHOM/BR/75/M2904 | 139 | 36.983

Phytomonas sp. isolate Hart1 | 97 | 36.7455

Leptomonas pyrrhocoris | 510 | 33.2548

Leptomonas seymouri | 34 | 31.6034

Trypanosoma conorhini | 89 | 31.3373

Mesangiospermae | 2 | 29.2666

Leishmania panamensis | 62 | 28.8435

Herpetomonas muscarum | 14 | 27.0233

##########

HR05 (VirNeg)

##########

TrypNT_cov | 425 | 2000.46

Leishmania donovani | 95 | 577.139

Trypanosoma | 684 | 324.802

Trypanosomatidae | 426 | 292.445

Leishmania | 278 | 206.722

Trypanosoma brucei | 2 | 56.6208

Eumetazoa | 1 | 42.2547

Trypanosoma theileri | 29 | 31.6385

Discoba | 4 | 30.9962

Leishmania major | 5 | 29.8326

Eukaryota | 111 | 28.2226

Leishmania mexicana (strain MHOM/GT/2001/U1103) | 7 | 20.4172

Capsicum baccatum | 1 | 19.5487

Leishmania tropica | 1 | 19.3703

Trypanosoma conorhini | 8 | 18.836

cellular organisms | 34 | 18.3457

root | 9 | 16.5593

Gammaproteobacteria | 1 | 16.5091

Lygus hesperus | 90 | 15.8575

Leishmania infantum | 2 | 14.7022

##########

B_raabi_123cor (VirNeg)

##########

TrypNT_cov | 3170 | 166.087

Trypanosoma | 1439 | 154.963

Leptomonas | 1131 | 141.104

Achromobacter sp. | 2 | 110.288

Trypanosomatidae | 694 | 89.1326

Crithidia sp. ATCC 30255 | 13 | 51.3263

Trypanosoma with unspecified subgenus | 9 | 50.0364

Trypanosoma brucei gambiense (strain MHOM/CI/86/DAL972) | 1 | 42.9069

Bodo saltans | 17 | 39.3741

Leptomonas pyrrhocoris | 641 | 39.0223

Leishmania braziliensis | 87 | 37.3415

root | 8 | 33.1387

Eukaryota | 52 | 32.4567

Crithidia acanthocephali | 12 | 29.0458

Leishmania infantum | 38 | 25.0601

Leishmania braziliensis MHOM/BR/75/M2904 | 62 | 23.7473

Leishmania tarentolae | 59 | 23.6293

Neobodo designis | 6 | 22.8727

Trypanosoma sp. TCC339 | 1 | 22.2488

Leishmania panamensis | 38 | 21.839

##########

CC37A (Qin)

##########

TrypNT | 457 | 974.119

Leptomonas pyrrhocoris qin-like virus | 1 | 524.133

Hubei qinvirus-like virus 2 | 1 | 333.878

Leptomonas pyrrhocoris leishbunyavirus 4 | 1 | 302.172

Leishmania enriettii | 3 | 37.0952

Phytomonas sp. EM1 | 1 | 36.03

Trypanosoma brucei | 1 | 10.0268

##########

CC49A (VirNeg)

##########

Trypanosomatidae | 560 | 736.495

Leishmania donovani | 309 | 630.975

TrypNT_cov | 2322 | 429.436

Trypanosoma | 1158 | 364.628

Leishmania | 1107 | 187.902

Trypanosoma grayi | 97 | 31.8254

Trypanozoon | 12 | 26.6397

Eukaryota | 46 | 23.4128

cellular organisms | 16 | 22.853

Xanthomonadaceae | 1 | 22.8457

Crithidia sp. ATCC 30255 | 14 | 22.7962

Leishmania panamensis | 39 | 22.468

Leptomonas pyrrhocoris | 400 | 22.2188

Leishmania tarentolae | 52 | 21.1723

Trypanosoma cruzi | 93 | 20.1524

Leptomonas | 554 | 18.8583

Trypanosoma brucei | 5 | 17.936

Leishmania braziliensis | 113 | 17.37

Trypanosoma theileri | 41 | 16.4735

Ophiostoma piceae (strain UAMH 11346) | 1 | 15.3331

##########

CV01 (partial Narna, low coverage)

##########

TrypNT_cov | 2956 | 466.776

Trypanosomatidae | 837 | 307.027

Trypanosoma | 1667 | 216.681

cellular organisms | 26 | 57.7789

Crithidia sp. ATCC 30255 | 7 | 57.7342

Mesangiospermae | 1 | 50.2264

Phytomonas sp. isolate Hart1 | 107 | 36.7679

Leptomonas pyrrhocoris | 473 | 31.0625

Leishmania infantum | 45 | 28.2594

Trypanosoma cruzi marinkellei | 11 | 27.6164

Crithidia acanthocephali | 11 | 27.264

Leptomonas | 660 | 25.8778

Leishmania panamensis | 43 | 24.4071

Lygus hesperus | 18 | 24.1901

Leishmania | 1295 | 24.0802

Trypanosoma brucei | 9 | 24.0754

Sordariomycetes | 2 | 24.0714

Eukaryota | 69 | 23.9496

Trypanosoma grayi | 133 | 22.5807

Leishmania donovani | 363 | 22.5767

##########

CV03 (Narna)

##########

Phytomonas serpens narnavirus 1 | 1 | 845.103

Eukaryota | 9 | 216.738

Trypanosomatidae | 73 | 187.904

Trypanosoma | 146 | 148.127

Bodo saltans | 4 | 134.205

Leishmaniinae | 45 | 130.784

Leishmania | 119 | 101.958

TrypNT_cov | 917 | 52.6789

Trypanosoma cruzi | 12 | 46.3411

Trypanosoma cruzi marinkellei | 1 | 34.8178

Trypanosoma grayi | 6 | 26.3574

Leptomonas | 57 | 25.9043

Leishmania donovani | 28 | 22.8963

Viannia | 1 | 17.7582

Leishmania braziliensis | 9 | 17.294

Trypanozoon | 1 | 16.6321

Leptomonas pyrrhocoris | 45 | 15.7677

Leishmania guyanensis | 1 | 15.2279

unclassified Phytomonas | 9 | 15.2122

Leishmania tarentolae | 8 | 15.188

Angomonas deanei | 13 | 12.2121

##########

CV26 (Narna, Mito)

##########

viral metagenome | 2 | 6332.71

Annperkins narna-like virus | 6 | 2826

Mitovirus sp. | 1 | 1704.32

unclassified Narnavirus | 5 | 1442.5

Trypanosomatidae | 1026 | 339.448

Trypanosoma | 1890 | 243.799

TrypNT_cov | 5265 | 136.766

Leishmania donovani | 483 | 132.058

Leishmania panamensis | 52 | 126.984

Crithidia sp. ATCC 30255 | 6 | 105.87

cellular organisms | 18 | 65.794

Leishmania | 1420 | 63.977

Scophthalmus maximus | 1 | 63.6298

Leptomonas | 695 | 58.1139

Lygus hesperus | 28 | 43.9719

Eukaryota | 72 | 38.5929

root | 4 | 38.3306

Trypanosoma congolense (strain IL3000) | 16 | 35.1955

Leishmania braziliensis MHOM/BR/75/M2904 | 119 | 33.9557

Leishmania braziliensis | 129 | 32.5357

##########

CV28A (VirNeg)

##########

Trypanosoma conorhini | 124 | 263.769

Leishmania donovani | 615 | 262.387

Trypanosoma rangeli | 232 | 136.028

TrypNT_cov | 5356 | 135.665

Trypanosomatidae | 1637 | 124.497

Leishmania | 1785 | 112.427

Trypanosoma | 3172 | 91.3736

Prymnesium parvum | 1 | 55.8455

Eukaryota | 50 | 49.4496

cellular organisms | 26 | 48.2329

Crithidia sp. ATCC 30255 | 5 | 46.8185

Ditylum brightwellii | 1 | 46.2398

Trypanosoma cruzi | 149 | 34.8256

Lygus hesperus | 20 | 29.5811

Leptomonas pyrrhocoris | 694 | 28.9155

Leishmania major | 169 | 26.6305

Herpetomonas muscarum | 12 | 25.0588

Leishmania braziliensis | 140 | 23.8441

Leptomonas | 1047 | 23.7083

Trypanosoma grayi | 157 | 23.6828

##########

M12 (VirNeg)

##########

TrypNT_cov | 2390 | 314.061

Entamoeba invadens IP1 | 1 | 295.963

Trypanosomatidae | 812 | 244.131

Trypanosoma | 1500 | 226.505

Leishmania donovani | 382 | 201.343

Entamoeba | 2 | 199.298

Leishmania | 1098 | 82.9499

Leishmania tropica | 4 | 64.5951

Dikarya | 1 | 39.2054

Trypanosoma conorhini | 56 | 28.4459

Leishmania braziliensis MHOM/BR/75/M2904 | 84 | 27.5128

Leishmania major | 75 | 26.2175

Trypanosoma grayi | 103 | 23.9656

Lygus hesperus | 27 | 23.3657

Leishmania braziliensis | 111 | 22.6526

cellular organisms | 25 | 20.5007

Leptomonas pyrrhocoris | 459 | 18.357

Kinetoplastea | 1 | 17.6573

Monosiga brevicollis | 3 | 16.4492

Herpetomonas muscarum | 10 | 15.5547

##########

PNG17 (VirNeg)

##########

Trypanosoma conorhini | 130 | 466.14

TrypNT_cov | 4148 | 246.367

Trypanosomatidae | 1205 | 166.984

Trypanosoma | 2454 | 164.492

Leishmania donovani | 510 | 68.0455

Leishmania | 1738 | 66.2592

Herpetomonas muscarum | 22 | 50.8723

Crithidia sp. ATCC 30255 | 13 | 40.9584

Lygus hesperus | 45 | 34.4771

Pseudozyma antarctica (strain T-34) | 1 | 34.1793

Leptomonas pyrrhocoris | 598 | 29.4222

Trypanosoma grayi | 160 | 29.2499

Leishmania braziliensis | 171 | 28.4533

Leishmania panamensis | 59 | 27.9088

Leishmania braziliensis MHOM/BR/75/M2904 | 156 | 27.1353

Pavlovaceae | 2 | 26.2818

Leishmania tarentolae | 67 | 25.3937

root | 9 | 22.8651

Leptomonas | 926 | 22.5394

Eukaryota | 78 | 22.0855

##########

p57 (VirNeg)

##########

TrypNT_cov | 756 | 302.314

Trypanosoma cruzi | 133 | 163.123

Trypanosomatidae | 949 | 111.538

Trypanosoma | 1736 | 85.7984

Leishmania donovani | 244 | 74.5215

Leishmania donovani species complex | 37 | 71.7922

Trypanosoma rangeli | 89 | 58.1823

Leishmania braziliensis | 46 | 53.6722

Tritrichomonas foetus | 1 | 52.4333

Leishmania | 620 | 38.7458

Leptomonas pyrrhocoris | 98 | 25.3175

Oceanobacter sp. | 1 | 24.8531

Trypanosoma theileri | 74 | 23.7014

Lygus hesperus | 192 | 19.2868

Metakinetoplastina | 389 | 18.5435

Trypanosoma congolense (strain IL3000) | 33 | 16.6069

Rozella allomycis (strain CSF55) | 2 | 16.1222

Leishmaniinae | 765 | 15.2593

Tetrahymena thermophila (strain SB210) | 4 | 15.2508

Viannia | 24 | 14.9327
