# Supplementary material for: Identification of diverse RNA viruses in Obscuromonas flagellates (Euglenozoa: Trypanosomatidae: Blastocrithidiinae)
Source: Virus Evol. 2024 May 4;10(1):veae037. doi: 10.1093/ve/veae037 (PMC11108086; doi:10.1093/ve/veae037)
Supplement: veae037_Supp [file veae037_supp.zip › suppl_data/Data S2.docx]

(OR192165.1_Sauroleishmania_gymnodactyli_LV247_narnavirus:0.78603867,((((Obscuromonas_CV08_narnavirus_1:0.00000236,Obscuromonas_CC37A_narnavirus_1:0.00000236)99:0.00000285,Obscuromonas_CV01_mitovirus_1:0.00000282)97:0.00700815,Obscuromonas_CV26_narnavirus_1:0.00000271)100:0.83092000,((((AYP67570.1__Wangarabell_virus:0.84262447,QPN36958.1__Praha_narnalike_virus_2:0.88413352)88:0.19030453,UYL95376.1__Qingyuan_Narna_tick_virus_1:0.76675417)94:0.18202617,(QIJ70074.1__Middlebrook_narnalike_virus:0.25245984,QNS17453.1__Serbia_narnalike_virus_2:0.17137110)100:0.67870716)57:0.14208958,(((((UJQ92833.1__Narnaviridae_sp.:0.56419828,(UGZ04787.1__Saccharomyces_20S_RNA_narnavirus:0.27071027,UQB84405.1__Saccharomyces_cerevisiae_narnavirus_3:0.44940820)100:0.34053207)93:0.28652769,QIJ70063.1__Tynnyfer_narnalike_virus:1.08694651)77:0.13685878,APG77120.1__Hubei_narnalike_virus_16:1.35818019)91:0.22231122,((QBC65281.1__Rhizopus_microsporus_23S_narnavirus:0.81372263,UHM27560.1__Sanya_narnavirus_10:1.18805450)60:0.10432706,UUW20991.1__Guiyang_narnalike_virus_1:1.10094510)69:0.18557373)83:0.21804547,(YP_009552002.1__Blechomonas_maslovi_narnavirus_1:1.82710375,(((((((Obscuromonas_CV22_narnavirus_1:0.00000272,Obscuromonas_CV03_narnavirus_1:0.00381072)100:0.21296341,AUF41955.1__Phytomonas_serpens_narnavirus_1:0.21665915)100:1.06295920,(UUG74247.1__XiangYun_narnalevilike_virus_14:0.25746289,YP_009553325.1__Leptomonas_seymouri_Narnalike_virus_1:0.39835817)100:0.54917167)61:0.13457751,((UFT26921.1__Drosophilaassociated_narnavirus_4:0.13955041,UFT26920.1__Drosophilaassociated_narnavirus_3:0.10443598)96:0.13801866,UFT26913.1__Drosophilaassociated_narnavirus_5:0.21611960)100:1.04582497)99:0.34310320,((UUG74246.1__XiangYun_narnalevilike_virus_13:0.43777148,YP_009333179.1__Beihai_barnacle_virus_10:0.61430861)100:0.33461708,((((AEM89293.1__Phytophthora_infestans_RNA_virus_4:0.17719776,QIP68008.1__Bremia_lactucae_associated_narnavirus_1:0.14751091)100:0.50123632,DAZ89879.1__Matryoshka_RNA_virus_4:0.47039109)58:0.11184620,(UUG74249.1__XiangYun_narnalevilike_virus_16:0.36308342,UHL62023.1__Narnavirus_sp.:0.41630986)100:0.17447028)100:0.47049406,USL98312.1__Phytophthora_castaneae_RNA_virus_2:0.85161149)91:0.15670807)90:0.23500316)96:0.47397663,((UUW20993.1__Guiyang_Paspalum_thunbergii_narnalike_virus_1:0.49247655,QIR30310.1__Plasmopara_viticola_lesion_associated_narnavirus_31:0.52467572)71:0.18929332,UAW09568.1__Aspergillus_flavus_narnavirus_2:0.55936588)100:1.47187651)95:0.54540735,(((UUW21051.1__Hangzhou_botourmialike_virus_6:0.70854560,QKI79959.1__Erysiphe_necator_associated_ourmialike_virus_130:0.27786092)100:1.02668559,((QDB75007.1__Phaeoacremonium_minimum_ourmialike_virus_2:0.67843787,(QGY72544.1__Plasmopara_viticola_lesion_associated_ourmialike_virus_14:0.66499343,QYC95708.1__Pestalotiopsis_botourmiavirus_1:0.92207839)64:0.19352322)46:0.13962090,UTQ50817.1__Plant_associated_botourmialike_virus_2:0.76266962)100:0.64100946)100:0.78395638,((((((AQM32767.1__Agaricus_bisporus_mitovirus_1:0.49252240,(QED55404.1__Heterobasidion_mitovirus_2:0.41123314,WEA82906.1__Armillaria_borealis_mitovirus_1:0.29594607)100:0.23085242)55:0.08546269,(QIR30256.1__Plasmopara_viticola_lesion_associated_mitovirus_33:0.09467801,QJX15645.1__Pea_associated_mitovirus_3:0.07114266)100:0.42416425)73:0.17999266,(QIJ70066.1__Annperkins_narnalike_virus:0.69813473,(((Obscuromonas_CV08_mitovirus_1b:0.00000236,Obscuromonas_CC37A_mitovirus_1b:0.00000278)87:0.00295222,Obscuromonas_CV22_mitovirus_1b:0.00144442)100:0.02357396,(Obscuromonas_CV08_mitovirus_1a:0.00000100,Obscuromonas_CV26_mitovirus_1a:0.00000100)100:0.01668182)100:0.77436524)75:0.20216061)22:0.04638850,((UUW21447.1__Guiyang_mitolike_virus_5:0.45009890,UUW21446.1__Guiyang_mitolike_virus_4:0.72670441)80:0.16343682,UUW21463.1__Guiyang_Paspalum_thunbergii_mitolike_virus_1:0.66588334)57:0.10176861)100:0.78196848,((((YP_005352912.1__Clitocybe_odora_virus:0.68183931,QED55409.1__Heterobasidion_mitovirus_3:0.62316600)96:0.28427314,(UIW13865.1__Rhizoctonia_solani_mitovirus_83:0.59256468,ALD89119.1__Rhizoctonia_solani_mitovirus_14:0.72519582)64:0.12918158)41:0.08327304,(AMM45292.1__Macrophomina_phaseolina_mitovirus_3:0.42812870,UIW13819.1__Rhizoctonia_solani_mitovirus_47:0.39254050)100:0.29257043)99:0.35393065,(((QXI69641.1__Rhizoctonia_solani_mitovirus_43:0.55261828,UIW13820.1__Rhizoctonia_solani_mitovirus_48:0.66126554)96:0.22954615,UIW13866.1__Rhizoctonia_solani_mitovirus_84:1.13211277)54:0.13854452,AWY10987.1__Sclerotinia_sclerotiorum_mitovirus_29:0.76785249)94:0.28476286)99:0.35616359)100:0.92716098,((((YP_010377187.1__Enterobacteria_phage_GA:0.46122823,AFN39800.1__Escherichia_phage_MS2:0.42834540)99:0.31241892,UJQ85776.1__Leviviridae_sp:0.43366812)95:0.34647969,APG77225.1__Hubei_levi-like_virus_5:0.77138836)89:0.45427628,NP_695028.1__Enterobacteria_phage_SP:0.64547172)100:2.34532077)77:0.38974582)72:0.24690086)72:0.15030136)94:0.42182768)94:0.23968150)100:0.68703733)94:0.23163012,(YP_009552755.1__Blechomonas_wendygibsoni_narnavirus_1:0.08277276,YP_009553634.1__Blechmonas_luni_narnavirus_1:0.15733251)100:0.52360149);
