# Supplementary material for: Identification of diverse RNA viruses in Obscuromonas flagellates (Euglenozoa: Trypanosomatidae: Blastocrithidiinae)
Source: Virus Evol. 2024 May 4;10(1):veae037. doi: 10.1093/ve/veae037 (PMC11108086; doi:10.1093/ve/veae037)
Supplement: veae037_Supp [file veae037_supp.zip › suppl_data/Data S3.docx]

(Obsc_CC37A_qin:0.881288,(((UYE93841.1__Hallarsbo:0.897532,(LeppyrUA_Zp02_qin-like_virus:0.004949,LeppyrSE_Sb07_qin-like_virus:0.000002)100:0.654610)97:0.254644,((((UYE93927.1__Ilomantsi:0.199625,QGA70919.1__Nackenback_virus:0.201974)100:0.197245,UUG74201.1__XiangYun:0.396534)100:0.180502,(UYE93931.1__Palkane:0.196713,(ASA47357.1__Wilkie_qin-like_virus:0.018940,(ASA47299.1__Wilkie_qin-like_virus:0.007389,ASA47455.1__Wilkie_qin-like_virus:0.003324)96:0.011981)100:0.206745)100:0.289000)100:0.645744,(YP_009337864.1__Wenzhou_qinvirus-like_virus_2:1.464765,((((((YP_009337847.1__Hubei_qinvirus-like_virus_1:0.586057,(YP_009342465.1__Wuhan_insect_virus_15:0.581115,AXA52552.1__Linepithema_humile_qinvirus-like_virus_1:0.648052)100:0.145271)95:0.103080,QNS31049.1__Frankliniella_occidentalis_associated_qin-like_virus1:1.172467)84:0.105981,QMP82146.1__Collembolan_qin-related_virus_OKIAV112:1.130603)100:0.358545,YP_009330273.1__Beihai_sesarmid_crab_virus_4:1.057211)84:0.153746,YP_009337866.1__Wenzhou_qinvirus-like_virus_1:1.372154)100:0.424265,((QIP68028.1__Plasmopara_viticola_lesion_associated_Yue-like_virus_2:2.130089,QIP68027.1__Plasmopara_viticola_lesion_associated_Yue-like_virus_1:2.428700)92:0.306867,(QIP68029.1__Plasmopara_viticola_lesion_associated_Yue-like_virus_3:2.152139,((YP_009337854.1__Shahe_yuevirus-like_virus_1:0.629688,YP_009330274.1__Beihai_sesarmid_crab_virus_3:1.051932)97:0.321860,QIP68011.1__Bremia_lactucae_associated_yuevirus-like_virus_1:0.685820)100:1.892910)91:0.288587)100:0.726592)97:0.158083)87:0.173921)100:0.533256)87:0.124865,((QLJ83493.1__Fitzroy_Crossing_qinvirus_1:0.084799,QGA70924.1__Vinslov_virus:0.107003)100:0.528171,(APG78066.1__Hubei_qinvirus-like_virus_2:0.581344,QGA70948.1__Vittskovle_virus:0.653198)50:0.083770)68:0.109828)80:0.135490,QGA70917.1__Gran_virus:0.954227);
