# Supplementary material for: Identification of diverse RNA viruses in Obscuromonas flagellates (Euglenozoa: Trypanosomatidae: Blastocrithidiinae)
Source: Virus Evol. 2024 May 4;10(1):veae037. doi: 10.1093/ve/veae037 (PMC11108086; doi:10.1093/ve/veae037)
Supplement: veae037_Supp [file veae037_supp.zip › suppl_data/Data S4.docx]

(Blastocrithidia_triatomae_TLV_EVE:0.4775301973,((KPA84471__Leptomonas_pyrrhocoris_TLV_EVE:0.5524849571,ASN64756__Leptomonas_pyrrhocoris_TLV:0.3686199059)64:0.1483081252,QZZ63393__Leuven_Tombus-like_virus_6:0.4565991872)56:0.1193285079,(QRW41690__Vai_augu_virus:0.7658304928,(((((((((AHA86931__Brandmavirus_UC1:0.6300429525,QDH88218__Riboviria_sp_:0.4153354793)37:0.1091035915,APG76615__Wenzhou_tombus-like_virus_17:0.4946653232)24:0.1157684281,((APG76457__Hubei_tombus-like_virus_36:0.3804517130,QDH91576__Riboviria_sp_:0.3337305780)29:0.0527672560,QDH89718__Riboviria_sp_:0.4367284093)24:0.0444775968)56:0.0933514143,APG76278__Changjiang_tombus-like_virus_22:0.4944140414)68:0.1065029175,(APG76480__Hubei_tombus-like_virus_35:0.3150044728,QCE20589__Melipona_quadrifasciata_virus_2:0.2565980290)100:0.2422681073)88:0.1832018490,USC30196__Tombusviridae_sp_:0.5867729983)68:0.1210315085,(YP_009344965__Jingmen_tombus-like_virus_2:0.4816806438,UCS96382__Riboviria_sp_:0.3784574226)100:0.6460413144)37:0.0419461812,(APG76183__Beihai_tombus-like_virus_17:1.0082868724,APG76207__Beihai_tombus-like_virus_18:0.5678515768)100:0.6108101626)57:0.1096523183,(((((((YP_009011225__Anopheline-associated_C_virus:0.4495414933,(AXA52557__Linepithema_humile_C_virus_1:0.3459166774,UDY81187__Chronic_bee_paralysis_virus:0.3798144655)60:0.1062574549)16:0.0378550055,QZZ63337__Leuven_wasp-associated_virus_1:0.3656191423)27:0.0520086867,(AKH40306__Dansoman_virus:0.2130360215,YP_009337686__Hubei_tombus-like_virus_42:0.2872484000)91:0.1528134128)58:0.0734945135,APG76342__Hubei_odonate_virus_12:0.5858338915)88:0.1162050861,(YP_009337115__Wenling_tombus-like_virus_4:0.2892335385,USC30270__Tombusvirus_sp_:0.3523363085)100:0.4027946082)84:0.1936538546,(((UDY80878__Lake_Sinai_virus_3:0.0578641328,UDY80974__Lake_Sinai_virus_4:0.0357563461)90:0.0524175563,UDY81018__Lake_Sinai_virus_2:0.0763469425)69:0.0553019087,UDY81090__Lake_Sinai_virus_8:0.0782515981)100:0.6025176462)100:0.4943803559,((((((CAB38331__Carnation_mottle_virus:0.3133939984,BAA92792__Japanese_iris_necrotic_ring_virus:0.4039684198)73:0.1222202974,AEC50092__Olive_mild_mosaic_virus:0.4537146132)100:0.2724601620,APG76248__Changjiang_tombus-like_virus_14:0.5874500867)96:0.2668836896,((QKN89005__Riboviria_sp_:0.1999239750,QUS52783__Mute_swan_feces_associated_tombus-like_virus_5:0.1837707427)100:0.2253102353,(QVG74799__Riboviria_sp_:0.3926877201,DAD57178__Riboviria_sp_:0.4183380815)99:0.1848315431)99:0.1880388210)50:0.0810291393,(((APG76308__Hubei_mosquito_virus_4:0.5491118971,UGO57611__Riboviria_sp_:0.3697587728)100:0.3171193055,(QTJ63611__Hemipteran_tombus-related_virus:0.6495099850,QTJ63617__Phasmatodean_tombus-related_virus:0.4679282173)76:0.0792330962)54:0.0862667115,(UCS96371__Riboviria_sp_:0.3866539922,UHM27700__Fushun_tombus-like_virus_1:0.6224260696)100:0.5779732685)80:0.1858275707)41:0.2005897859,YP_009342294__Wenzhou_tombus-like_virus_10:0.9244270450)100:0.4139551689)98:0.3658201328)98:0.3833714360)49:0.0585046414);
