# Supplementary figures and images for: Identification of diverse RNA viruses in Obscuromonas flagellates (Euglenozoa: Trypanosomatidae: Blastocrithidiinae)
Source: Virus Evol. 2024 May 4;10(1):veae037. doi: 10.1093/ve/veae037 (PMC11108086; doi:10.1093/ve/veae037)

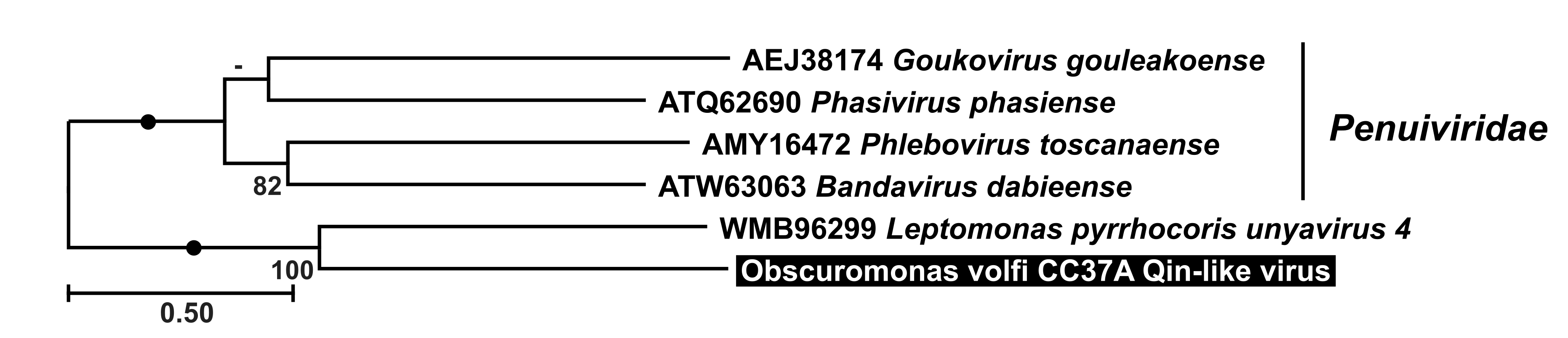

Supplement: veae037_Supp [file veae037_supp.zip › suppl_data/Fig S1 R1.png]
